# Blocks in cycles and *k*-commuting permutations

**DOI:** 10.1186/s40064-016-3638-7

**Published:** 2016-11-10

**Authors:** Rutilo Moreno, Luis Manuel Rivera

**Affiliations:** 1 Instituto de Física, Universidad Autónoma de San Luis Potosí, San Luis Potosí, Mexico; 2 Fakultät Für Mathematik, Universität Wien, Oskar-Morgenstern-Platz 1, 1090 Wien, Austria; 3 UAIE and UAM, Universidad Autónoma de Zacatecas, Calzada Solidaridad entronque Paseo a la Bufa, 98000 Zacatecas, Mexico

**Keywords:** Symmetric group, Hamming metric, Commutation relation, Enumeration, Primary 05A05; Secondary 05A15, 20B30

## Abstract

We introduce and study *k*-commuting permutations. One of our main results is a characterization of permutations that *k*-commute with a given permutation. Using this characterization, we obtain formulas for the number of permutations that *k*-commute with a permutation $\beta$, for some cycle types of $\beta$. Our enumerative results are related with integer sequences in “The On-line Encyclopedia of Integer Sequences”, and in some cases provide new interpretations for such sequences.

## Background

The symmetric group as a metric space has been studied with different metrics and for different purposes (see, e.g., Deza and Huang 1998; Diaconis 1988; Farahat 1960), and the metric that seems to be more used is the Hamming metric. This metric was introduced by Hamming (1950) for the case of binary strings and in connection with digital communications. For the case of permutations, it was used by Farahat (1960), who studied the symmetries of the metric space $(S_n, H)$, where $S_n$ denotes the symmetric group on the set $\{1, \dots ,n\}$ and *H* the Hamming metric between permutations. Also, Gorenstein et al. (1962) studied a problem about permutations that almost commute, in the sense of normalized Hamming metric.

Other problems are showed in the survey of Quistorff (2006), about the packing and covering problem, and in the survey of Cameron (2010), about permutation codes. This last problem have turned out to be useful in applications to power line communications, as was showed in Chu et al. (2004).

In this paper, we introduce and study *k*-commuting permutations. It seems that this is the first time this issued is studied. Shallit (2009) worked in an slightly similar problem but with strings. One of our main results is a characterization of the permutations that *k*-commute with a given permutation $\beta$. This characterization is given in terms of blocks formed by strings of points in cycles in the decomposition of $\beta$ as a product of disjoint cycles.

Our original motivation to study this type of questions was to develop tools to work with problems related with the stability of the commutator relator in permutations. Recently, Arzhantseva and Păunescu (2015) proved that the equation $xy=yx$ is stable in permutations. The concept of stability of equations in permutations appears recently in Glebsky and Rivera (2009), in the context of sofic groups, that is a class of groups of growing interest that was defined by Gromov (1999) [details about sofic groups can be consulted in the monograph of Ceccherini-Silberstein and Coornaert (2010) or in the survey of Pestov (2008)].

The analogous problem about the stability of $xy=yx$ in matrices is a classical problem in linear algebra and operator theory, and has been widely studied (see, e.g., Friis and Rørdam 1996; Hastings 2009; Lin 1997; Voiculescu 1983; Filonov and Safarov 2011; Glebsky 2010).

In some cases, we need to know upper bounds for the number of permutations that almost commute with a given permutation, as in Păunescu (2016). With this in mind, we work in the problem of determine the number $c(k,\beta )$ of permutations that *k*-commute with $\beta$. In this paper, we present explicit formulas for $c(k,\beta )$, when $\beta$ is any permutation and $k\le 4$. The study of this small cases sheds light of how difficult it can be the problem of computing $c(k, \beta )$ in its generality. So, we have worked with several specific types of permutations. Surprisingly, we have found some relations between $c(k, \beta )$ and the following integer sequences in the OEIS database of Sloane (2015): A208529, A208528 and A098916 when $\beta$ is a transposition, A000757 when $\beta$ is an *n*-cycle (this relationship allows us to obtain the binomial transform of sequence A000757), and A053871 when $\beta$ is a fixed-point free involution.

The relationship between the number $c(k, \beta )$ with some integers sequences in the OEIS database have provided another motivation to studied this problem. Using the techniques developed in this paper, Rivera (2015) showed more such relationship, and also identities between integer sequences in the OEIS database.

We review our results. We present a characterization of permutations that *k*-commute with a given permutation $\beta$. This characterization is given in terms of blocks in cycles in the decomposition of $\beta$ as a product of disjoint cycles. Also, we present a formula and a bivariate generating function for the number of permutations that *k*-commute with any *n*-cycle. We present explicit formulas for the number $c(k, \beta )$, when $\beta$ is any permutation and $k\le 4$. Finally, we present formulas for the cases when $\beta$ is either a transposition or a fixed-point free involution. In all cases, we present relationship between our formulas and sequences in the OEIS database.

## Definitions and notation

We first give some definitions and notation used throughout the work. The elements in the set $[n]:=\{1, \dots , n\}$ are called *points*. We write $\pi =p_1 p_2 \dots p_n$ for the one-line notation of $\pi \in S_n$, i.e., $\pi (i)=p_i$ for every $i \in [n]$. We compute the product $\alpha \beta$ of permutations $\alpha$ and $\beta$ by first applying $\beta$ and then $\alpha$. It is a known fact that any permutation can be written in essentially one way as a product of disjoint cycles, called its *cycle decomposition* (Dummit and Foote 2004, Sec. 1.3, p. 29). We say that $\pi$ *has cycle* $\pi '$ or that $\pi '$ is *a cycle of* $\pi$ if $\pi '$ is a cycle in the disjoint cycle factorization of $\pi$. Let $\pi '=(a_1 \dots a_m)$ be a cycle of $\pi$, we use $\mathrm{set}(\pi ')$ to denote the set $\{a_1, \dots , a_m\}$. We say that *a* is a point in cycle $\pi '$ if $a \in \mathrm{set}(\pi ')$. The *cycle type* of a permutation $\beta$ is a vector $(c_1, \dots , c_n)$ such that $\beta$ has exactly $c_i$ cycles of length *i* in its cycle decomposition. The *Hamming metric* between permutations $\alpha , \beta \in S_n$, denoted $H(\alpha , \beta )$, is $|\{ a \in [n] :\alpha (a) \ne \beta (a)\}|$ [see the survey of Deza and Huang (1998) for more details about this metric]. It is well-known that this metric is bi-invariant, that not two permutations have Hamming metric equal to 1, and that $H(\alpha , \beta )=2$ if and only if $\alpha \beta ^{-1}$ is a transposition. We say that $a\in [n]$ is a *good commuting point* (resp. *bad commuting point*) of $\alpha$ and $\beta$ if $\alpha \beta (a)= \beta \alpha (a)$ (resp. $\alpha \beta (a)\ne \beta \alpha (a)$). Usually, we abbreviate good commuting points (resp. bad commuting points) with *g.c.p.* (resp. *b.c.p.*). In this work, we use the convention $m \bmod m = m$ for any positive integer *m*.

### Blocks in cycles

Our definition of blocks was motivated by the definitions presented in the work of Christie (1996) and in the work of Bóna and Flynn (2009). A *block* *A* in a cycle $\pi '=(a_1a_2\dots a_m)$ of $\pi \in S_n$ is a consecutive nonempty substring $a_ia_{i+1} \dots a_{i+l}$, of $a_i\dots a_{i-1}$ where $(a_i\dots a_{i-1})$ is one of the *m* equivalent expressions of $\pi '$ (the sums are taken modulo *m*). The *length* |*A*| of block $A=a_i \dots a_{i+l}$ is the number of elements in the string *A*, and the points $a_i$ and $a_{i+l}$ are the *first* and the *last* element of the block, respectively. A *proper block* (resp. *improper block*) of an *m*-cycle is a block of length $l < m$ (resp. $l=m$). Two blocks *A* and *B* are *disjoint* if they do not have points in common. The *product* *AB* of two disjoint blocks, *A* and *B*, not necessarily from the same cycle of $\pi$, is defined as the usual concatenation of strings (*AB* is not necessarily a block in a cycle of $\pi$). If $(a_1\dots a_m)$ is a cycle of $\pi$ we write $(A_1\dots A_k)$ to mean that $A_1\dots A_k=a_i\dots a_{i-1}$, where $(a_i \dots a_{i-1})=(a_1\dots a_m)$. A *block partition* of cycle $\pi '$ is a set $\{A_1, \dots , A_l\}$ of pairwise disjoint blocks in $\pi '$ such that there exist a block product $A_{i_1}\dots A_{i_l}$ of these blocks such that $\pi '=(A_{i_1}\dots A_{i_l})$. Let $p= J_{1}J_{2}\dots J_{k}$ be a block product of *k* pairwise disjoint blocks, not necessarily from the same cycle of $\pi$, and let $\tau$ be a permutation in $S_k$. The *block permutation* $\phi _\tau (P)$ of *P*, induced by $\tau$, is defined as the block product $J_{\tau (1)}J_{\tau (2)}\dots J_{\tau (k)}$.

#### *Example 1*

Let $\pi =(1\;2\;3\;4)(5\;6\;7\;8\;9)\in S_9$. Some blocks in cycles of $\pi$ are $P_1=2\;3\;4\;1$ and $P_2=1\;2$, $B_1=5\;6\;7$, $B_2=8$, $B_3=9$. The set $\{B_1, B_2, B_3\}$ is a block partition of $(5\;6\;7\;8\;9)$. The product $B_1B_2$ is a block in $(5\;6\;7\;8\;9)$. The product $P_2B_2=128$ is not a block in any cycle of $\pi$. Let $\tau =(3\;1\;2) \in S_3$, then $\phi _\tau (B_1B_2B_3)=B_2B_3B_1=8\;9\;5\;6\;7$.

The restriction function of $\pi \in S_n$ to set $X \subseteq [n]$ is denoted by $\pi |_X$. Let $\alpha , \beta \in S_n$. Let $\beta '=(b_1\dots b_m)$ be a cycle of $\beta$. It is well known (Dummit and Foote 2004, Prop. 10, p. 125) that $\alpha \beta '\alpha ^{-1}=(\alpha (b_1) \dots \alpha (b_m))$. From this, we use the following matrix notation for $\alpha |_{\mathrm{set}(\beta ')}$

1 $$\begin{aligned} \alpha |_{\mathrm{set}(\beta ')}=\left( \begin{array}{ccc} b_1 &{} \dots &{} b_m \\ \alpha (b_1) &{} \dots &{} \alpha (b_m) \end{array} \right) . \end{aligned}$$

Notice that for a given cycle $\beta '$, there are *m* ways to write $\alpha |_{\mathrm{set}(\beta ')}$ in this matrix notation. If $\alpha |_{\mathrm{set}(\beta ')}$ is written as in (1), we write

$$\begin{aligned} \alpha |_{\mathrm{set}(\beta '), k}=\left( \begin{array}{c} B_1 \dots B_k \\ J_1 \dots J_k \end{array} \right) , \end{aligned}$$

to mean that $B_1 \dots B_k=b_1\dots b_m$, and $J_1, \dots , J_k$ are blocks in cycles of $\beta$, where $J_1 \dots J_k=\alpha (b_1)\dots \alpha (b_m)$ and $|J_i|=|B_i|$, for $1\le i \le k$. This notation is called a *block notation* (with respect to $\beta$) of $\alpha |_{\mathrm{set}(\beta ')}$. This notation depends on the particular selection of one of the *m* equivalent cyclic expressions of $\beta$. Sometimes we omit *k* in $\alpha |_{\mathrm{set}(\beta '), k}$, when *k* is clear.

#### *Example 2*

Let $\alpha , \beta \in S_6$, where $\alpha =(1\;3\;4)(2\;5\;6)$ and $\beta =(1\;2\;4\;5)(3\;6)$. If $\beta '=(1\;2\;4\;5)$, then $\alpha (1\;2\;4\;5)\alpha ^{-1}=(3\;5\;1\;6)$, and $\alpha |_{\mathrm{set}(\beta ')}$ can be written as

$$\begin{aligned} \alpha |_{\mathrm{set}(\beta ')}=\left( \begin{array}{cccc} 1 &{} 2 &{} 4 &{} 5 \\ 3 &{} 5 &{} 1 &{} 6 \end{array} \right) . \end{aligned}$$

Two ways of written $\alpha |_{\mathrm{set}(\beta ')}$ in block notation are

$$\begin{aligned} \alpha |_{\mathrm{set}(\beta '), 3}=\left( \begin{array}{|c|cc|c|} 1 &{} 2 &{} 4 &{} 5 \\ 3 &{} 5 &{} 1 &{} 6 \end{array} \right) , \quad \alpha |_{\mathrm{set}(\beta '), 4}=\left( \begin{array}{|c|c|c|c|} 1 &{} 2 &{} 4 &{} 5 \\ 3 &{} 5 &{} 1 &{} 6 \end{array} \right) , \end{aligned}$$

where the vertical lines denote the limits of the blocks.

## Permutations that *k*-commute with a cycle

In this section, we show the relation between blocks in cycles of $\beta$ and the permutations that *k*-commute with $\beta$.

Let $\beta '$ be a cycle of $\beta$. Let $\alpha$ be a permutation. If $\alpha \beta '\alpha ^{-1}$ is also a cycle of $\beta$, then we say that $\alpha$ *transforms* the cycle $\beta '$ into the cycle $\alpha \beta '\alpha ^{-1}$. Let $B=b_1 \dots b_l$ be a block in $\beta '$. We say that permutation $\alpha$ *commutes* with $\beta$ *on the block* *B* if $\alpha \beta (b_i)=\beta \alpha (b_i)$, for every $i \in \{1, \dots , l \}$. We say that $\alpha$ *commutes* (resp. do not commute) with $\beta$ on $\beta '$, if $\alpha \beta (b)=\beta \alpha (b)$, for every $b \in \mathrm{set}(\beta ')$ (resp. $\alpha \beta (b) \ne \beta \alpha (b)$, for some $b \in \mathrm{set}(\beta ')$).

The following result is the key to relate commutation and blocks in cycles.

### **Proposition 1**

*Let* $\alpha , \beta \in S_n$. *Let* $\ell , m$ *be integers, with* $1 \le \ell < m$. *Let* $\beta '=(b_1 \; \dots \; b_m)$ *be a cycle of* $\beta$. *If* $\alpha$ *commutes with* $\beta$ *on the block* $b_1 \dots b_\ell$, *then* $\alpha (b_1)\dots \alpha (b_\ell )\alpha (b_{\ell +1})$ *is a block in a cycle of* $\beta$.

### *Proof*

It is enough to prove that $\alpha (b_{i})=\beta ^{i-1}(\alpha (b_1))$, for $i \in \{1, \dots , \ell +1\}$. The proof is by induction on *i*. The base case $i=1$ is trivial. Assume as inductive hypothesis that the statement is true for every $k <\ell +1$. As $\alpha$ and $\beta$ commute on $b_k$, then $\alpha (b_{k+1})=\alpha (\beta (b_{k}))=\beta (\alpha (b_{k}))$, and by the inductive hypothesis, $\beta (\alpha (b_{k}))=\beta (\beta ^{k-1}(\alpha (b_1)))=\beta ^{k}(\alpha (b_1))$ as desired. $\square$

The following result is an easy exercise.

### **Proposition 2**

*Let* $\beta '$ *be an* *m*-*cycle of* $\beta$. *Then* $\alpha$ *commutes with* $\beta$ *on* $\beta '$ *if and only if* $\alpha$ *transforms* $\beta '$ *into an* *m*-*cycle of* $\beta$.

Let $\beta '$ be a cycle of $\beta$. We say that $\alpha (r, \beta )$-*commutes* with $\beta '$ if there exists exactly *r* points in $\beta '$ on which $\alpha$ and $\beta$ do not commute.

We now present one of our main results.

### **Theorem 1**

*Let* $\beta '$ *be an* *m*-*cycle of* $\beta$ *and* *k* *an integer such that* $k\ge 1$. *Then* $\alpha$ $(k, \beta )$-*commutes with* $\beta '$ *if and only if* $\alpha \beta '\alpha ^{-1}=(P_1\dots P_k)$, *where the blocks* $P_1, \dots , P_k$ *satisfy the following*:

1. *if* $k=1$, *then* $P_1$ *is a proper block in a cycle of* $\beta$;
2. *if* $k >1$, *then* $P_1, \dots , P_k$ *are* *k* *pairwise disjoint blocks, from one or more cycles of* $\beta$, *such that the string* $P_iP_{i+1\bmod k }$ *is not a block in any cycle of* $\beta$, *for every* $i \in [k]$.

### *Proof*

(1) Suppose that $\alpha \beta '\alpha ^{-1}=(P_1)$ where $P_1$ is a proper block in a cycle of $\beta$. Without lost of generality assume that $\beta '=(b_1 \dots b_m)$ and $P_1=\alpha (b_1) \dots \alpha (b_m)$. As $P_1$ is a block in a cycle of $\beta$, then $\beta (\alpha (b_i))=\alpha (b_{i+1})$, for $i \in \{1, \dots , m-1\}$, which implies that $\beta (\alpha (b_i))=\alpha (\beta (b_i))$, for $i \in \{1, \dots , m-1\}$. Finally, as $P_1$ is an improper block in a cycle of $\beta$, then $\beta (\alpha (b_m)) \ne \alpha (b_1)$, which implies that $\beta (\alpha (b_m)) \ne \alpha (\beta (b_m))$. Therefore $\alpha$ $(1, \beta )$-commutes with $\beta '$.

Conversely, we can assume, without lost of generality, that $\alpha$ commutes with $\beta$ on block $b_1 \dots b_{m-1}$ of $\beta '=(b_1 \dots b_{m-1}b_m)$ and that does not commute on $b_m$. By Proposition 1, $\alpha (b_1) \dots \alpha (b_{m-1})\alpha (b_m)$ is a block in a cycle of $\beta$ and it is a proper block due to Proposition 2.

(2) Suppose that $\alpha \beta ' \alpha ^{-1}=(P_1 \dots P_k)$, where every $P_i=p_{i1}p_{i2} \dots p_{i\ell _i}$ is a block in a cycle of $\beta$ and that for every $i \in [k]$, $P_iP_{i+1 \bmod k}$ is not a block in any cycle of $\beta$. Without lost of generality we can write the cycle $\beta '$ as

$$\begin{aligned} \beta '=(b_{11} \dots b_{1\ell _1} b_{21} \dots b_{2\ell _2} \dots b_{k1} \dots b_{k\ell _k}), \end{aligned}$$

with $\alpha (b_{ir})=p_{ir}$, for every $i \in [k]$ and $1\le r \le \ell _i$. As $P_i$ is a block in a cycle of $\beta$, then $\beta (p_{ir})=p_{i(r+1)}$, for every $r \in \{1, \dots , \ell _i-1\}$. Therefore, we have, for one side that $p_{i(r+1)}=\beta (p_{ir})=\beta (\alpha (b_{ir}))$, and for the other side $p_{i(r+1)}=\alpha (b_{i(r+1)})=\alpha \left( \beta (b_{ir})\right)$ which means that $\alpha$ and $\beta$ commute on $b_{ir}$, for every $r \in \{1, \dots , \ell _i-1\}$. We now prove that $\beta (\alpha (b_{i\ell _i}))\ne \alpha (\beta (b_{i\ell _i}))$. Suppose that $\beta (\alpha (b_{i\ell _i}))=\alpha (\beta (b_{i\ell _i}))$, as $\beta (p_{i\ell _i})=\beta (\alpha (b_{i\ell _i}))$, then $\beta (p_{i\ell _i})=\alpha (\beta (b_{i\ell _i}))=\alpha (b_{(i+1 \bmod k)1})=p_{(i+1 \bmod k)1}$, which implies that $P_iP_{i+1 \bmod k}$ is a block in a cycle of $\beta$, a contradiction.

Conversely, if $\alpha$ does not commute with $\beta$ on exactly *k* points in $\mathrm{set}(\beta ')$, then we can write $\beta '$ as $(B_1 \dots B_k)$, where $B_i=b_{i1}b_{i2} \dots b_{i\ell _i}$ is a block in a cycle of $\beta$, for every $i\in \{1, \dots , k\}$, and in this block, $\alpha$ and $\beta$ commute on $b_{ij}$, for $1\le j <\ell _i$, and does not commute on $b_{i\ell _i}$. By Proposition 1, we have that $P_i:=\alpha (b_{i1})\alpha (b_{i2}) \dots \alpha (b_{i\ell _i})$ is a block in a cycle of $\beta$. Now, suppose that for some *i*, $P_iP_{i+1 \bmod k}$ is a block in a cycle of $\beta$, then $\beta (\alpha (b_{i\ell _i}))=\alpha (b_{(i+1 \bmod k)1})=\alpha (\beta (b_{i\ell _i}))$, contradicting the assumption that $\alpha$ does not commute with $\beta$ on $b_{i\ell _i}$. $\square$

### *Remark 1*

The function $\alpha |_{\mathrm{set}(\beta '), k}$, using the notation from above, can be written as

$$\begin{aligned} \alpha |_{\mathrm{set}(\beta '), k}=\left( \begin{array}{c} B_1 \dots B_k \\ P_1 \dots P_k \end{array} \right) , \end{aligned}$$

with $\beta '=(B_1 \dots B_k)$, $\alpha \beta '\alpha ^{-1}=(P_1 \dots P_k)$. Where, for every $i \in \{1,\dots , k\}$, $|B_i|=|P_i|=\ell _i$, $B_i=b_{i1}b_{i2} \dots b_{i\ell _i}$, $P_i=p_{i1}p_{i2} \dots p_{i\ell _i}$, i.e., $\alpha (b_{ir})=p_{ir}$, with $1\le r\le \ell _i$; and $\alpha$ and $\beta$ commute on $b_{i1}b_{i2} \dots b_{i(\ell _i-1)}$ and do not commute on $b_{i\ell _i}=\alpha ^{-1}(p_{i\ell _i})$ (the last point of $B_i$).

Using Theorem 1 we can characterize permutations that *k*-commute with $\beta$.

### **Corollary 1**

*Let* $\alpha , \beta \in S_n$. *Then* $\alpha$ *k*-*commutes with* $\beta$ *if and only if there exist* *h* *cycles of* $\beta$, *say* $\beta _1, \dots , \beta _h$, *such that* $\alpha$ *commutes with* $\beta$ *on each cycle of* $\beta$ *not in* $\{\beta _1, \dots , \beta _h\}$ *and for every* $i \in \{1, \dots , h\}$, $\alpha \beta _i\alpha ^{-1}=(P_1^{(i)}\dots P^{(i)}_{k_i})$, *with* $k_i\ge 1$, $k=k_1+\dots +k_h$, *where the blocks* $P_1^{(i)}, \dots , P^{(i)}_{k_i}$ *satisfy the following*:

1. if $k_i=1$, then $P^{(i)}_1$ is a proper block in a cycle of $\beta$,
2. if $k_i >1$, then $P^{(i)}_1, \dots , P^{(i)}_{k_i}$ are $k_i$ pairwise disjoint blocks, from one or more cycles of $\beta$, such that $P^{(i)}_rP^{(i)}_{r+1\bmod k_i }$ is not a block in any cycle of $\beta$, for any $r \in [k_i]$.
3. $\{P_1^{(1)}, \dots , P_{k_1}^{(1)}, \dots ,P_1^{(h)}, \dots , P_{k_h}^{(h)}\}$ is a set of pairwise disjoints blocks from one or more cycles of $\beta$.

### *Example 3*

Let $\alpha , \beta \in S_7$, where $\beta =(1\;2\;4\;5\;3)(7\;6)$ and $\alpha =(2\;7)(3\;6\;4\;5)$. By direct calculations we can check that $\alpha$ $(4, \beta )$-commutes with $\beta _1=(1\;2\;4\;5\;3)$ (the b.c.p. are 1, 2, 3 and 5) and $(1, \beta )$-commutes with $\beta _2=(7\;6)$ (the b.c.p. is 6). In block notation, $\alpha |_{\mathrm{set}(\beta _1)}$ and $\alpha |_{\mathrm{set}(\beta _2)}$ can be written as

$$\begin{aligned} \alpha |_{\mathrm{set}(\beta _1), 4}=\left( \begin{array}{|c|c|cc|c|} 1 &{} 2 &{} 4 &{} 5 &{} 3 \\ 1 &{} 7 &{} 5 &{} 3 &{}6 \end{array}\right) , \quad \alpha |_{\mathrm{set}(\beta _2), 1}=\left( \begin{array}{|cc|} 7 &{} 6 \\ 2 &{} 4 \end{array}\right) . \end{aligned}$$

As a first application of Theorem 1, we obtain the following result, that is used in the proof of Theorem 8

### **Proposition 3**

*Let* $\beta$ *be a permutation whose maximum cycle length in its cycle decomposition is* *m*. *If* $\alpha$ *commutes with* $\beta$ *on* $m-1$ *points in an* *m*-*cycle* $\beta '$ *of* $\beta$, *then* $\alpha$ *commutes with* $\beta$ *on* $\beta '$.

### *Proof*

Suppose that $\alpha$ and $\beta$ do not commute on the remaining point in $\beta '$. By part (1) of Theorem 1, $\alpha \beta '\alpha ^{-1}=(P)$, where *P* is a proper block in an *l*-cycle of $\beta$, i.e., $l > m$, but this is a contradiction because *m* is the maximum cycle length of cycles in $\beta$. $\square$

Previous propositions is a generalization of Lemma 2(b) in Gorenstein et al. (1962). The following proposition will be useful in the proofs of some of our results.

### **Proposition 4**

*Let* $\alpha$ *and* $\beta$ *be two permutations that* *k*-*commute*, $k>0$. *Suppose that* $\alpha$ *does not commute with* $\beta$ *on the cycles* $\beta _1, \dots , \beta _r$, *of lengths* $l_1, \dots , l_r$, *respectively, and that commutes with* $\beta$ *on the rest of cycles of* $\beta$ *(if any). Then, there exists exactly* *r* *cycles of* $\beta$, *say* $\beta _1', \dots , \beta _r'$, *of lengths* $l_1, \dots l_r$, *respectively, such that* $\alpha \left( \mathrm{set}(\beta _1) \cup \dots \cup \mathrm{set}(\beta _r)\right) =\mathrm{set}(\beta _1') \cup \dots \cup \mathrm{set}(\beta _r')$. Even *more, suppose that* $\alpha$ *does not commute with* $\beta$ *on exactly* $h_i$ *i*-*cycles of* $\beta$ *and that commutes with* $\beta$ *on the rest of the* *i*-*cycles of* $\beta$ *(if any). Then there exists exactly* $h_i$ *i*-*cycles of* $\beta$ *such that each of them contains at least one point that is the image under* $\alpha$ *of one b.c.p. of* $\alpha$ *and* $\beta$.

### *Proof*

Let $\beta _{r+1}, \dots , \beta _{s}$ the rest of cycles of $\beta$ of lengths $l_{r+1}, \dots l_s$, respectively. As $\alpha$ commutes with $\beta$ on every one of this cycles, then $\alpha$ transforms each $\beta _{t}$ into an $l_t$-cycle $\beta _t'$, with $r+1 \le t \le s$ (by Proposition 2). Then, there are cycles of $\beta$, say $\beta _{r+1}', \dots , \beta _{s}'$, of lengths $l_{r+1}, \dots , l_s$, respectively, such that $\alpha \left( \mathrm{set}(\beta _{r+1}) \cup \dots \cup \mathrm{set}(\beta _s)\right) =\mathrm{set}(\beta _{r+1}') \cup \dots \cup \mathrm{set}(\beta _s')$, and the result of the first part of the proposition follows because $\alpha$ is a bijection.

By a similar argument, we can show that if $\alpha$ does not commute with $\beta$ on exactly $h_i$ *i*-cycles of $\beta$ and commutes with $\beta$ on the rest of the *i*-cycles of $\beta$ (if any), then there exists exactly $h_i$ *i*-cycles of $\beta$, say $\beta _1', \dots , \beta _{h_i}'$, such that $\beta _t'\ne \alpha \beta _j \alpha ^{-1}$, for every $t \in \{1, \dots , h_i\}$ and every cycle $\beta _j$ of $\beta$. The following claim completes the proof of the second part

### **Claim 1**

*If all the points in an* *i*-cycle $\beta _{1}$ *of* $\beta$ *are images under* $\alpha$ *of g.c.p., then there exists an* *i*-cycle $\beta _2$ *of* $\beta$ *such that* $\beta _{1}=\alpha \beta _2 \alpha ^{-1}$.

### *Proof*

First, we prove, by contradiction, that if all the points in the *i*-cycle $\beta _{1}$ are images under $\alpha$ of g.c.p. of $\alpha$ and $\beta$, then these g.c.p. belong to exactly one *l*-cycle, say $\beta _2=(b_1 \dots b_l)$, of $\beta$, with $l \ge i$. Suppose that $\beta _{1}$ contains the images under $\alpha$ of g.c.p. in different cycles of $\beta$, then $\beta _{2}$ contains the string $\alpha (x)\alpha (y)$, with *x* and *y* in different cycles of $\beta$, i.e., $\beta (x) \ne y$, but this implies that *x* is a b.c.p. because $\alpha (\beta (x))\ne \alpha (y)= \beta (\alpha (x))$. It is clear that $l \ge i$. Now, we show that $l \le i$. Suppose that $l >i$, then, and without lost of generality, we have that $\beta _2=(\alpha (b_1)\dots \alpha (b_i))$, i.e., $\beta (\alpha (b_t))=\alpha (b_{t+1 \bmod i})$, for every $t \in \{1, \dots , i\}$ (if $\beta (\alpha (b_t)) \ne \alpha (b_{t+1 \bmod i})$, for some *t*, then $b_t$ will be a b.c.p. of $\alpha$ and $\beta$). But this implies that $b_i$ is a b.c.p. because, for one side, $\beta (\alpha (b_i))=\alpha (b_1)$, and for the other side, $\alpha (b_1) \ne \alpha (b_{i+1})=\alpha (\beta (b_i))$ (as $l > i$, then $i+1 \bmod l \ne 1$ and hence $b_1 \ne b_{i+1}$) which is a contradiction. Therefore $l \le i$, and then $l=i$, i.e., $\beta _{1}= \alpha \beta _2\alpha ^{-1}$. $\square$

With this claim, every cycle in $\{\beta _1', \dots , \beta _{h_i}'\}$ contains at least one point that is the image under $\alpha$ of one b.c.p. of $\alpha$ and $\beta$ as desired. $\square$

We finish this subsection with the following result

### **Theorem 2**

*Let* $\alpha , \beta \in S_n$. *If one cycle of* $\beta$ *has exactly one b.c.p. of* $\alpha$ *and* $\beta$, *then there exist a cycle of* $\beta$ *that contains at least two b.c.p. of* $\alpha$ *and* $\beta$.

### *Proof*

Let $\beta _1$ be a cycle of $\beta$ that has exactly one b.c.p of $\alpha$ and $\beta$. The proof is by induction on the length *l* of cycle $\beta _1$. If $\beta _1$ is an 1-cycle, then, by Proposition 4, there exists an 1-cycle $\beta _2$ of $\beta$ that fixed a point, say *x*, such that $x'=\alpha ^{-1}(x)$ is a b.c.p. of $\alpha$ and $\beta$. From Proposition 2 it follows that $x'$ is a point in a cycle $\beta _{3}$ of $\beta$ of length greater than one. That is, $\beta _{3}$ is a cycle of the form $(x'B)$, with *B* a block of length $|B|\ge 1$. Therefore, $\alpha \beta _{3} \alpha ^{-1}=\left( xB'\right)$, and by Theorem 1, $\beta _{3}$ has at least two b.c.p. of $\alpha$ and $\beta$.

Now we consider the case $l >1$. Let $\beta _1=(d_1 \dots d_l)$ be a cycle of $\beta$ with exactly one b.c.p. of $\alpha$ and $\beta$. Without lost of generality we can suppose that $d_l$ is such a b.c.p. Assume as induction hypothesis that the statement of the proposition is true for *r*-cycles of $\beta$ which contains exactly one b.c.p of $\alpha$ and $\beta$ with $r <l$ (notice that in general, it could be the case that no such cycles in $\beta$ exists). Let ${\mathcal {C}}_l$ denote the set of all *l*-cycles of $\beta$ and $c_l=|{\mathcal {C}}_l|$. Let

$$\begin{aligned} \mathrm{set}({\mathcal {C}}_l)=\bigcup _{\beta ' \in {\mathcal {C}}_l}\mathrm{set}(\beta '). \end{aligned}$$

By Theorem 1, we have that $\alpha \beta _1\alpha ^{-1}=(D)$, where $D=\alpha (d_1) \dots \alpha (d_l)$ is a proper block in an *s*-cycle of $\beta$, with $s >l$. As $\alpha (d_1)$ does not belong to an *l*-cycle of $\beta$, then $d_1 \not \in \alpha ^{-1}(\mathrm{set}({\mathcal {C}}_l))$, but $d_1 \in \mathrm{set}({\mathcal {C}}_l)$, so we have that

$$\begin{aligned} \alpha ^{-1}\left( \mathrm{set}({\mathcal {C}}_l)\right) \ne \mathrm{set}({\mathcal {C}}_l). \end{aligned}$$

Therefore, there exist at least one *l*-cycle, say $\beta _2=(a_1\dots a_l)$, of $\beta$ (with the possibility that $\beta _2=\beta _1$) with at least one point, says $a_1$, which has its preimage under $\alpha$ in one *m*-cycle, say $\beta _3$, with $m \ne l$. Let *r* be an integer between 1 and *l* such that $\alpha ^{-1}(a_1) \dots \alpha ^{-1}(a_r)$ is a block in cycle $\beta _3=(b_1 \dots b_{m})$, with $b_i=\alpha ^{-1}(a_i)$, for $1\le i \le r$, but $\alpha ^{-1}(a_1) \dots \alpha ^{-1}(a_r)\alpha ^{-1}(a_{r+1 \bmod l})$ is not a block in $\beta _3$. We have the following cases:

- *Case I.* If $m >r$, then $\beta _3$ has at least two b.c.p. of $\alpha$ and $\beta$ (by Theorem 1).
- *Case II.* If $m=r$, then $r < l$ (because $m \ne l$ and $1\le r \le l$) and then $\alpha \beta _3 \alpha ^{-1}=(a_1 \dots a_m)$. As $a_1 \dots a_m$ is a proper block in $\beta _2$, then $\beta _3$ has exactly one b.c.p. of $\alpha$ and $\beta$ (by Theorem 1), and by the inductive hypothesis it follows that $\beta$ has a cycle with at least two b.c.p. of $\alpha$ and $\beta$.

$$\square $$

### Permutations that $(k, \beta )$-commute with a cycle of $\beta$

Let $\beta \in S_n$ be a fixed permutation and $k \ge 3$ be a positive integer. Let $\alpha$ be any permutation that *k*-commutes with $\beta$ and that $(k, \beta )$-commutes with an *m*-cycle, say $\beta _1$, of $\beta$, i.e., $m \ge k$ and all the b.c.p. of $\alpha$ and $\beta$ are in $\beta _1$. From Proposition 4 it follows that there exists exactly one *m*-cycle, say $\beta _{2}$, of $\beta$ such that $\mathrm{set}(\beta _{2})=\alpha (\mathrm{set}(\beta _1))$. Using this fact we present a procedure (Algorithm 1) that allows us to obtain any such permutation $\alpha$. First we give some definitions. The *canonical cycle notation* of a permutation $\pi$ is defined as follows: first, write the largest element of each cycle, and then arrange the cycles in increasing order of their first elements. Let $\pi$ be a permutation written in its canonical cycle notation, the *transition function* of $\pi$ from canonical cycle notation to one-line notation is the map $\Psi : S_n \rightarrow S_n$ that sends $\pi$ to the permutation $\Psi (\pi )$ written in one-line notation that is obtained from $\pi$ by omitting all the parentheses. This map is a bijection (Bóna 2004, p. 97).

#### *Example 4*

Let $\pi \in S_7$ be $(4\;3\;1)(6\;5)(7\;2)$ ($\pi$ is written in its canonical cycle notation). Then $\Psi (\pi )=4316572$.

#### **Algorithm 1**

Step 1: Choose two *m*-cycles, say $\beta _{1}$ and $\beta _{2}=(p_1 \dots p_m)$, of $\beta$ (with the possibility that $\beta _{2}=\beta _1$).
Step 2: Choose a *k*-subset $\{p_{h_1}, \dots , p_{h_k}\}$ of $\mathrm{set}(\beta _{2})$. Without lost of generality suppose that $p_{h_k}=p_m$, and that $h_1< \dots < h_k$. Now, to make a block partition $\{P_1, \dots , P_k\}$ of $P=p_1 \dots p_m$ as follows
  $$\begin{aligned} \underbrace{p_1 \dots p_{h_1}}_{P_1}\underbrace{p_{h_1+1} \dots p_{h_2}}_{P_2} \dots \underbrace{p_{h_{k-1}+1} \dots p_{h_k}}_{P_k}. \end{aligned}$$
  Notice that $p_{h_r}$ is the last point of $P_r$, $1\le r \le k$.
Step 3: Choose a *k*-cycle permutation $\tau =(i_1 \dots i_k)$ of $[k]=\{1, \dots ,k\}$ such that $\tau (a) \ne a+1 \bmod k$, for every $a\in [k]$, and make the block permutation
  $$\begin{aligned} P':=P_{\Psi (\tau )(1)} P_{\Psi (\tau )(2)} \dots P_{\Psi (\tau )(k)}=P_{i_1} P_{i_2} \dots P_{i_k}. \end{aligned}$$
Step 4: Construct $\alpha |_{\mathrm{set}(\beta _1)}: \mathrm{set}(\beta _1) \rightarrow \mathrm{set}(\beta _{2})$ as it follows:
  $$\begin{aligned} \alpha |_{\mathrm{set}(\beta _1), k}=\left( \begin{array}{c} B_1 B_2 \dots B_k \\ P_{i_1} P_{i_2} \dots P_{i_k} \end{array} \right) . \end{aligned}$$
  where $\beta _1=(B_1\dots B_k)$ and $|B_r|=|P_{i_r}|$, for every $r \in \{1, \dots , k\}$.
Step 5: Construct $\alpha |_{[n] \setminus \mathrm{set}(\beta _1)} :[n] \setminus \mathrm{set}(\beta _1) \rightarrow [n] \setminus \mathrm{set}(\beta _{2})$ as any bijection that commutes with $\beta |_{[n] \setminus \mathrm{set}(\beta _1)} :[n] \setminus \mathrm{set}(\beta _1) \rightarrow [n] \setminus \mathrm{set}(\beta _{2})$.

Let $c_m$ be the number of *m* cycles of $\beta$. For Step 5, $\alpha$ can be constructed in such a way that it transforms the $c_m-1$ *m*-cycles of $\beta$ different than $\beta _1$ (if any) into the $c_m-1$ *m*-cycles of $\beta$ different than $\beta _{2}$ (if any), and that transforms the *l*-cycles of $\beta$ (if any), with $l \ne m$, into *l*-cycles of $\beta$ (if any).

The following two propositions shows that Algorithm 1 produces all the permutations with the desired properties.

#### **Proposition 5**

*Any permutation* $\alpha$ *constructed with Algorithm* 1 *does not commute with* $\beta$ *on all points in* ${\mathcal {A}}:=\alpha ^{-1}(\{p_{h_1},\dots , p_{h_k}\})$ *and commutes with* $\beta$ *on all points in* $[n] \setminus {\mathcal {A}}$.

#### *Proof*

Let $\beta _1$ and $\beta _{2}$ be the cycles of $\beta$ selected in Step 1 of Algorithm 1, and $\{p_{h_1}, \dots , p_{h_k}\}$ the subset of $\mathrm{set}(\beta _{2})$ selected in Step 2. By the way in which $\alpha$ is constructed of in Step 3 and 4, $\alpha \beta _1 \alpha ^{-1}=(P_{i_1} P_{i_2} \dots P_{i_k})$, where $P_{i_r}P_{i_{r+1 \bmod k}}$, is not a block in any cycle of $\beta$ (by Step 3, $i_{r+1 \bmod k} - i_r \bmod k \ne 1$), $1\le r \le k$. From Theorem 1, we have that $\alpha$ does not commute with $\beta$ on exactly *k* points in $\mathrm{set}(\beta _1)$. Even more, in the proof of Theorem 1 was showed that $\alpha$ and $\beta$ do not commute on $\alpha ^{-1}(p_{h_r})$, for $r\in \{1, \dots , k\}$ (see Remark 1). Finally, by the construction of $\alpha$ in Step 5, $\alpha$ and $\beta$ commute on all points in $[n] \setminus \mathrm{set}(\beta _1)$. $\square$

#### **Proposition 6**

*Let* $k \ge 3$. *Let* $\alpha$ *be any permutation that* *k*-*commutes with* $\beta$ *and such that all the b.c.p. of* $\alpha$ *and* $\beta$ *are in exactly one* *m*-*cycle of* $\beta$. *Then* $\alpha$ *can be obtained with Algorithm* 1.

#### *Proof*

Let $\beta _1$ be the *m* cycle of $\beta$ that has all the b.c.p. of $\alpha$ and $\beta$. From Proposition 4 it follows that there exists exactly one *m*-cycle, $\beta _{2}$, of $\beta$ such that $\alpha (\mathrm{set}(\beta _1))=\mathrm{set}(\beta _{2})$. By Theorem 1, we have that $\alpha \beta _1 \alpha ^{-1}=(P_1 \dots P_k)$, where $P_{1}, \dots , P_{k}$ are *k* pairwise disjoint blocks in $\beta _{2}$ and $P_{r} P_{r+1 \mod k}$ is not a block in any cycle of $\beta$, for every $r \in \{1, \dots , k\}$. As $\alpha (\mathrm{set}(\beta _1))=\mathrm{set}(\beta _{2})$, we have that $P_{1} \dots P_{k}$ is a block permutation of $B'=P_{i_1} \dots P_{i_k}$, where $\beta _{2}=(B')$. Now, rename the blocks $P_{i_s}$ as $B'_s$ to obtain $B'=B'_1 \dots B'_k$. In this way, $\alpha \beta _1 \alpha ^{-1}=(B'_{l_1}\dots B'_{l_k})$, with $l_{r+1 \bmod k}-l_r \bmod k \ne 1$, for every $r \in \{1, \dots , k\}$. Indeed, if $l_{r+1 \bmod k}-l_r \bmod k= 1$ for some $r \in \{1, \dots , k\}$, then $B'_{l_r}B'_{l_{r+1 \bmod k}}$ will be a block in $\beta _{2}$, and hence the number of b.c.p. of $\alpha$ and $\beta$ will be less than *k*, which is a contradiction.

As $\alpha \beta _1\alpha ^{-1}=(B'_{l_1} \dots B'_{l_k})=(B'_{l_2} \dots B'_{l_1})=\dots =(B'_{l_k} \dots B'_{l_{k-1}})$, we can assume without lost of generality that $l_1=k$ (from these *k* expressions, choose the one that begins with block $B_k'$). Then $\alpha |_{\mathrm{set}(\beta _1)}$ can be written as

$$\begin{aligned} \alpha |_{\mathrm{set}(\beta _1), k}=\left( \begin{array}{c} B_1 \dots B_k \\ B'_{l_1} \dots B'_{l_k} \end{array} \right) , \end{aligned}$$

where $\beta _1=(B_1\dots B_k)$, and $|B_i|=|B'_{l_i}|$, with $1\le i \le k$.

Now, we consider $l_1 \dots l_{k}$ as a permutation, named $\pi$, of $\{1, \dots , k\}$ in one-line notation. As $l_1$ (that is equal to *k*) is the greatest element in $\{l_1, \dots , l_k\}$, then $\tau :=\Psi ^{-1}(\pi )=(l_1 \dots l_{k})$, where $\Psi$ is the transition function from the canonical cycle notation to one-line notation. Notice that $\tau$ is a *k*-cycle in $S_k$ such that $\tau (a) \ne a+1$, for any $a \in [k]$. Thus we conclude that $\alpha |_{\mathrm{set}(\beta _j)}$ can be obtained by Steps 1–4 of Algorithm 1. As $\alpha$ commutes with $\beta$ on all cycles different than $\beta _j$, $\alpha |_{[n]\setminus \mathrm{set}(\beta _j)}$ can be obtained with Step 5 of Algorithm 1. $\square$

## On the number $c(k, \beta )$

In this section we present some results about the number $c(k, \beta )$ of permutations that *k*-commute with $\beta$. Let $C_{S_n}(\beta )$ denote the centralizer of $\beta$. Let $C(k, \beta )$ be the set $\{\alpha \in S_n :H(\alpha \beta , \beta \alpha )=k\}$, then $c(k, \beta )=|C(k, \beta )|$.

### **Proposition 7**

*Let* $\beta \in S_n$ *be a permutation of cycle type* $(c_1, \dots , c_n)$. *Then* $c(0, \beta )=\prod _{i=1}^{n}i^{c_i}c_i!$, *and* $c(1,\beta )=c(2,\beta )=0$.

### *Proof*

When $k=0$, $c(0,\beta )$ is the size of the centralizer of $\beta$. As no two permutations have Hamming metric equal to 1 then $c(1, \beta )=0$. Finally, it is easy to see that $H(\pi , \tau )=2$ if and only if $\pi \tau ^{-1}$ is a transposition. If $H(\alpha \beta , \beta \alpha ) = 2$ then the even permutation $\alpha \beta \alpha ^{-1}\beta ^{-1}$ should be a transposition which is a contradiction. $\square$

Now we show that for any nonnegative integer *k* and any $\beta \in S_n$, the number $c(k, \beta )$ is invariant under conjugation.

### **Proposition 8**

*If* $\beta \in S_n$, *then* $c(k, \tau \beta \tau ^{-1}) =c(k, \beta )$, *for any* $\tau \in S_n$.

### *Sketch of the proof*

For $$\tau \in S_n$$, let

$$\begin{aligned} \tau C(k, \beta ) \tau ^{-1}=\{\tau \alpha \tau ^{-1} :\alpha \in C(k, \beta )\}. \end{aligned}$$

By the bi-invariance of the Hamming metric, is straightforward to show that $C(k, \tau \beta \tau ^{-1})=\tau C(k, \beta ) \tau ^{-1}$. Now, it is easy to check that the function $\phi : C(k, \beta ) \rightarrow \tau C(k, \beta ) \tau ^{-1}$ given by $\sigma \mapsto \tau \sigma \tau ^{-1}$ is a bijection. Therefore, $|C(k, \beta )|=|\tau C(k, \beta ) \tau ^{-1}|= | C(k, \tau \beta \tau ^{-1})|$. $\square$

The following result shows that $c(k, \beta )$ is a multiple of $|C_{S_n}(\beta )|$.

### **Proposition 9**

*Let* $\beta \in S_n$. *Suppose that* $C(k,\beta )$ *is a non-empty set. Then*

$$\begin{aligned} C(k,\beta )=\bigcup _{\alpha \in C(k, \beta )}C_{S_n}(\beta )\alpha . \end{aligned}$$

### *Proof*

Now, let $\rho \in \bigcup _{\alpha \in C(k, \beta )}C_{S_n}(\beta )\alpha$, then $\rho = \tau \alpha$ for some $\tau \in C_{S_n}(\beta )$ and some $\alpha \in C(k, \beta )$. So we have that

$$\begin{aligned} H(\rho \beta ,\beta \rho )=H(\tau \alpha \beta ,\beta \tau \alpha )=H(\tau \alpha \beta ,\tau \beta \alpha )=H(\alpha \beta ,\beta \alpha )=k, \end{aligned}$$

and then $\rho \in C(k, \beta )$. The other inclusion is clear. $\square$

### On the number $c([k], \beta )$

Let $c([k], \beta )$ denotes the number of permutations $\alpha$ that *k*-commutes with $\beta$ which satisfy the extra condition that all the b.c.p. of $\alpha$ and $\beta$ are in exactly one cycle of $\beta$.

Let *f*(*k*) be the number of cyclic permutations (*k*-cycles) of $\{1, \dots , k\}$ with no $i \mapsto i+1\bmod k$ (Stanley 1997, exercise 8, p. 88). Sequence $\{f(k)\}$ is labeled as A000757 in the OEIS database.

#### **Theorem 3**

*Let* $\beta \in S_n$ *be a permutation of cycle type* $(c_1, \dots , c_n)$. *Let* *k* *be an integer, with* $3 \le k \le n$. *Then*

$$\begin{aligned} c(\lambda _{[k]}, \beta ) = |C_{S_n}(\beta )| \sum _{\ell \ge k}^n c_\ell \left( {\begin{array}{c}\ell \\ k\end{array}}\right) f(k). \end{aligned}$$

#### *Proof*

As all the b.c.p. of $\alpha$ and $\beta$ are in one $\ell$-cycle, say $\beta _1=(b_1\dots b_\ell )$, of $\beta$, with $\ell \ge k$, then the images under $\alpha$ of the b.c.p are in exactly one $\ell$-cycle, say $\beta _{2}=(b_1'\dots b_\ell ')$, of $\beta$ (by Proposition 4). There are $$\ell \left( {\begin{array}{c}\ell \\ k\end{array}}\right) f(k)$$ ways to construct a bijection $\alpha |_{\mathrm{set}(\beta _1)}:\mathrm{set}(\beta _1) \rightarrow \mathrm{set}(\beta _{2})$ with steps 2 to 4 in Algorithm 1. Indeed, there are $$\left( {\begin{array}{c}\ell \\ k\end{array}}\right) $$ ways to choose the subset in Step 2; there are *f*(*k*) ways to select the permutation $\tau$ in Step 3, and there are $\ell$ ways to select the first point in block $B_1 \dots B_k$ in Step 4. Now, let $c_\ell$ denotes the number of $\ell$-cycles in the cycle decomposition of $\beta$. There are $c_\ell ^2$ ways to select the $\ell$-cycles $\beta _1$ and $\beta _{2}$, there are $(c_\ell -1)!\ell ^{c_\ell -1}$ ways to make that $\alpha$ transforms the $c_\ell -1$ cycles of length $\ell$ of $\beta$ different than $\beta _1$ into the $c_\ell -1$ cycles of $\beta$ of length $\ell$ different than $\beta _{2}$. Now for every $t \ne \ell$ there are $t^{c_t}c_t!$ ways to make that $\alpha$ transforms all the $c_t$ *t*-cycles of $\beta$ into $c_t$ *t*-cycles of $\beta$ and hence there are $\frac{1}{\ell ^{c_\ell }c_\ell !}|C_{S_n}(\beta )|$ ways to make that $\alpha$ transforms all the cycles of length $t \ne \ell$ into cycles of length $t \ne \ell$. Finally, we sum over all possible lengths $\ell \ge k$ of cycles of $\beta$. Therefore

$$\begin{aligned} c([k], \beta )&=\sum _{\ell \ge k}^n \ell \left( {\begin{array}{c}\ell \\ k\end{array}}\right) f(k) c_\ell ^2 (c_\ell -1)!\ell ^{c_\ell -1}\frac{1}{\ell ^{c_\ell }c_\ell !}|C_{S_n}(\beta )|\\ &=|C_{S_n}(\beta )| \sum _{\ell \ge k}^n c_\ell \left( {\begin{array}{c}\ell \\ k\end{array}}\right) f(k). \end{aligned}$$

$$\square $$

Let *T*(*k*, *n*) denote the number of *n*-permutations that *k*-commute with an *n*-cycle.

#### **Corollary 2**

*Let* *n* *be a positive integer and* *k* *and integer with* $0 \le k \le n$. *Then*

$$\begin{aligned} T(k, n) = n\left( {\begin{array}{c}n\\ k\end{array}}\right) f(k). \end{aligned}$$

The number *T*(*k*, *n*) is now sequence A233440 in the OEIS database. With this corollary we can obtain, in an easy way, the binomial transform of sequence A000757. Let $A=\{f(0), f(1), \dots \}$ be sequence A000757, and let $B=\{b_0, b_1, \dots \}$ be the binomial transform of *A*. In Spivey and Steil (2006), $b_n$ is defined as $$\sum _{k=0}^n \left( {\begin{array}{c}n\\ k\end{array}}\right) f(k)$$. By Corollary 2, $b_n=\sum _{k=0}^n T(k, n)/n$. As $\sum _{k=0}^nT(k, n)=n!$, then $b_n=(n-1)!$.

We have the following limit property for *T*(*k*, *n*).

#### **Proposition 10**

*Let* *n* *be a positive integer and* *m* *be a fixed nonnegative integer with* $m\ne n$. *Then*

$$\begin{aligned} \lim _{n \rightarrow \infty } \frac{T(n-m,\; n)}{n!}=\frac{e^{-1}}{m!}. \end{aligned}$$

#### *Proof*

By direct calculations we have that

$$\begin{aligned} \frac{T(n-m, n)}{n!}= \frac{f(n-m)}{m!(n-m-1)!}+\frac{mf(n-m)}{m!(n-m)(n-m-1)!}, \end{aligned}$$

The result follows by using that $\lim _{k \rightarrow \infty } f(k)/(k-1)!=e^{-1}$ (Stanley 1997, exercise 8-e, p. 88). $\square$

#### **Theorem 4**

*Let* *n*, *k* *be positive integers with* $k \le n$. *Then*

$$\begin{aligned} \sum _{n,k} T(k, n)\frac{z^n}{n!}u^k=ze^{z(1-u)}\left( \left( 1-\log (1-zu)\right) \left( 1-u\right) +\frac{u}{1-zu}\right) . \end{aligned}$$

#### *Proof*

Let $g^{\langle k \rangle }(z)=\sum _n g_{n, k} \frac{z^n}{n!}$ denotes the vertical generating function (exponential case) of the sequence $\{g_{n, k}\}$. Let $$c_{n, k}:= T(k, n)/n=\left( {\begin{array}{c}n\\ k\end{array}}\right) f(k)$$. From Example 3. 1, in (Flajolet and Sedgewick 2009, p. 155), and by using the fact that function *f*(*k*) is independent of *n* we have

$$\begin{aligned} c^{\langle k \rangle }(z)=\sum _n \left( {\begin{array}{c}n\\ k\end{array}}\right) f(k)\frac{z^n}{n!}=f(k) \frac{e^zz^k}{k!}. \end{aligned}$$

Now, by using Rule (2’) in (Wilf 1994, p. 41) we obtain

$$\begin{aligned} \sum _n n \left( {\begin{array}{c}n\\ k\end{array}}\right) f(k)\frac{z^n}{n!}=f(k) z\left( \frac{e^zz^k}{k!}+\frac{e^zz^kk}{zk!}\right) . \end{aligned}$$

Now

$$\begin{aligned} P(z, u)&:=   \sum _{k, n}n\left( {\begin{array}{c}n\\ k\end{array}}\right) f(k) \frac{z^n}{n!}u^k\\ &=  \sum _k \left( \sum _n n\left( {\begin{array}{c}n\\ k\end{array}}\right) f(k)\frac{z^n}{n!}\right) u^k\\&=  \sum _k f(k) z\left( \frac{e^zz^k}{k!}+\frac{e^zz^kk}{zk!}\right) u^k\\ &= \sum _k f(k) z\frac{e^zz^k}{k!}u^k+\sum _k f(k) z\frac{e^zz^kk}{zk!}u^k \\ &=   ze^z\sum _k f(k) \frac{z^ku^k}{k!}+e^z\sum _k k f(k) \frac{z^ku^k}{k!}. \end{aligned}$$

It is known that $\sum _{k\ge 0} f(k) \frac{x^k}{k!}=e^{-x}(1-\log (1-x))$ (Stanley 1997, exercise 8, p. 88), then

$$\begin{aligned} ze^z\sum _k f(k) \frac{z^ku^k}{k!}=ze^z(e^{-zu}(1-\log (1-zu)). \end{aligned}$$

Now, we apply Rule (2’) in Wilf (1994) to the second term of *P*(*z*, *u*) to obtain

$$\begin{aligned} e^z\sum _k k f(k) \frac{z^ku^k}{k!}=e^z(zu)e^{-zu}\left( \frac{1}{1-zu}-(1-\log (1-zu))\right) , \end{aligned}$$

and the result follows after some algebraic manipulations. $\square$

## The number $c(k, \beta )$ for $k=3, 4$

In this section we present formulas for the number $c(k, \beta )$, when $\beta$ is any permutation of cycle type $(c_1, \dots , c_n)$ and $k=3, 4$. We use the following notation: Let $[k_1, \dots , k_h]$ denote an integer partition of *k*, with $k_i \ge 1$. We define a set $C([k_1, \dots , k_h], \beta )$ as follows: $\alpha \in C([k_1, \dots , k_h], \beta )$ if and only if $\alpha$ *k*-commutes with $\beta$, and there are exactly *h* cycles, says $\beta _1, \dots , \beta _h$, in $\beta$, such that $\alpha$ $(k_1, \beta )$-commutes with $\beta _1$, $(k_2, \beta )$-commutes with $\beta _2$, ..., $(k_h, \beta )$-commutes with $\beta _h$. Let $c([k_1, \dots , k_h], \beta )$ be the cardinality of $C([k_1, \dots , k_h], \beta )$. By Theorem 2, we have that $c([1, \dots , 1], \beta )=0$, where $[1, \dots , 1]$ denotes the partition of *k* that consists of *k* ones.

### **Theorem 5**

*Let* $\beta$ *be any* *n*-*permutation of cycle type* $(c_1, \dots , c_n)$. *Then*

$$\begin{aligned} c(3, \beta )=\left( \sum _{\ell \ge 3}^n c_\ell \left( {\begin{array}{c}\ell \\ 3\end{array}}\right) +\sum _{1 \le \ell <m\le n} \ell mc_\ell c_m\right) |C_{S_n}(\beta )|. \end{aligned}$$

### *Proof*

The number $c(3, \beta )$ is equal to $c([1, 1, 1], \beta )+c([2, 1], \beta )+c([3], \beta )$. The case $c([3], \beta )$ follows from Theorem 3 and $c([1, 1, 1], \beta )=0$. To obtain $c([2, 1], \beta )$, we construct all permutations $\alpha$ that 3-commute with $\beta$ and such that $\beta$ has a unique $\ell$-cycle (resp. *m*-cycle), say $\beta _1$ (resp. $\beta _2$), where $\alpha$ ($1, \beta$)-commutes with $\beta _1$ (resp. $(2, \beta )$-commutes with $\beta _2$). By Proposition 4, there exist exactly one $\ell$-cycle $\beta '_1$ of $\beta$ and exactly one *m*-cycle $\beta '_2$ of $\beta$ such that $\alpha \left( \mathrm{set}(\beta _1) \cup \mathrm{set}(\beta _2)\right) =\mathrm{set}(\beta _1') \cup \mathrm{set}(\beta _2')$. From Theorem 1 we have that

2 $$\begin{aligned} \alpha |_{\mathrm{set}(\beta _1)\cup \mathrm{set}(\beta _2)}=\left( \begin{array}{c} A_1 \\ X_1 \end{array}\right) \left( \begin{array}{cc} B_1 &{} B_2 \\ X_2 &{}X_3 \end{array}\right) ; \end{aligned}$$

where

- $\beta _1=(A_1)$, $\beta _2=(B_1B_2)$, $X_2, X_3$ are blocks distributed in $\beta '_1$ and $\beta '_2$, and $X_1$ is a block in a cycle of length greater that $A_1$, i.e., $X_1$ is a block in $\beta '_2$;
- the strings $X_2X_3$ and $X_3X_2$ are not blocks in any cycle of $\beta$,
- The set of all points in the blocks $X_1, X_2, X_3$ is equal to $\mathrm{set}(\beta '_1)\cup \mathrm{set}(\beta '_2)$.

From condition (a) to condition (c) we have that $X_2$ and $X_3$ belongs to different cycles. Without lost of generality we can assume that $\beta _1'=(X_2)$ and that $\beta _2'=(X_1X_3)$. Now we count the number of ways to construct $\alpha |_{\mathrm{set}(\beta _1)\cup \mathrm{set}(\beta _2)}$. There are $\ell$ ways to select the first point of block $A_1$ and there are *m* ways to select the first point of block $B_1B_2$. There are $\ell$ ways to select the first point of block $X_2$ and there are *m* ways to select the first point of block $X_1$ (after this selection, the first point of block $X_3$ is uniquely determined).

There are $c_\ell ^2c_m^2$ ways to select the $\ell$-cycles and *m*-cycles $\beta _1, \beta '_1$ and $\beta _2, \beta '_2$. There are $(c_\ell -1)!\ell ^{c_\ell -1}(c_m-1)!m^{c_m-1}$ ways to make that $\alpha$ transforms the $c_\ell -1$ cycles of length $\ell$ of $\beta$ different that $\beta _1$ into the $c_\ell -1$ cycles of length $\ell$ of $\beta$ different than $\beta '_1$ and the $c_m-1$ cycles of length *m* of $\beta$ different that $\beta _2$ into the $c_m-1$ cycles of length *m* of $\beta$ different than $\beta '_2$. Now, for every $t \not \in \{m, \ell \}$ there are $t^{c_t}c_t!$ ways to make that $\alpha$ transforms all the $c_t$ *t*-cycles of $\beta$ into $c_t$ *t*-cycles of $\beta$ and hence there are $\frac{1}{m^{c_m}c_m!\ell ^{c_\ell }c_\ell !}|C_{S_n}(\beta )|$ ways to make that $\alpha$ transforms all the cycles of length different than $\ell$ and *m* into cycles of length different than $\ell$ and *m*. After summing over all possible values of $\ell$ and *m* we have that $c([2, 1], \beta )$ is equal to

$$\begin{aligned}&\sum _{1 \le \ell<m\le n} (\ell m)^2c_\ell ^2c_m^2 (c_\ell -1)!\ell ^{c_\ell -1}(c_m-1)!m^{c_m-1}\frac{1}{m^{c_m}c_m!\ell ^{c_\ell }c_\ell !}|C_{S_n}(\beta )|\\&\quad =\sum _{1 \le \ell <m\le n} \ell mc_\ell c_m|C_{S_n}(\beta )|. \end{aligned}$$

$$\square $$

In a similar way, but with many more cases to consider, we have obtained a formula for $c(4, \beta )$, for any $\beta$. In order to avoid an unnecessarily increase in the length of this paper, we have omitted the proof but the interested reader can consulted it in the preprint version of this paper (Moreno and Rivera 2014).

### **Theorem 6**

*Let* $\beta$ *be any permutation of cycle type* $(c_1, \dots , c_n)$. *Then*

$$\begin{aligned} c(4, \beta )=c(\lambda _{4^{(1)}}, \beta ) +c([3, 1], \beta )+c([2,2], \beta )+c([2, 1,1], \beta ), \end{aligned}$$

*where*

$$\begin{aligned} c(\lambda _{4^{(1)}}, \beta ) &=  |C_{S_n}(\beta )| \sum _{i \ge 4}c_i \left( {\begin{array}{c}i\\ 4\end{array}}\right) ;\\ c([3, 1], \beta )&=  |C_{S_n}(\beta )| \sum _{i \ge 1, j \ge i+2} ij(j-i-1)c_ic_j;\\ c([2, 2], \beta )&=  |C_{S_n}(\beta )|\left( \sum _{i\ge 2}i\left( {\begin{array}{c}i\\ 2\end{array}}\right) \left( {\begin{array}{c}c_i\\ 2\end{array}}\right) +\sum _{j>i\ge 2}i(i-1)jc_ic_j\right) ;\\ c([2, 1,1], \beta )&= |C_{S_n}(\beta )|\left( \sum _{i \ge 1} i^3c_{2i}\left( {\begin{array}{c}c_i\\ 2\end{array}}\right) + \sum _{j>i\ge 1}ij(i+j)c_ic_jc_{i+j} \right) . \end{aligned}$$

## Transpositions and fixed-point free involutions

In this section we show formulas for $c(k, \beta )$ when $\beta$ is either a transposition or a fixed-point free involution. Let $\mathrm {fix}(\beta )$ denotes the set of fixed points of $\beta$ and $\mathrm {supp}(\beta )=[n] {\setminus} \mathrm {fix}(\beta )$.

### **Proposition 11**

*Let* $\alpha , \beta \in S_n$ *and let* $H(\alpha \beta , \beta \alpha )=k$, *then* $0 \le k \le 2 | \mathrm {supp}(\beta )|$.

### *Proof*

If $\alpha$ commutes with $\beta$, then $k=0$. If $\beta$ does not have fixed points then $|\mathrm {supp}(\beta )|=n$ and $k < 2|\mathrm {supp}(\beta )|$. Now, let $x \in \mathrm {fix}(\beta )$. If $\beta \alpha (x) \ne \alpha \beta (x)$ then $\alpha (x) \in \mathrm {supp}(\beta )$ (Theorem 1). Thus, $\alpha$ does not commute with $\beta$ on at most $| \mathrm {supp}(\beta )|$ fixed points of $\beta$ and then $k \le 2 | \mathrm {supp}(\beta )|$. $\square$

The following theorem is a consequence of Proposition 7, Theorem 5, Theorem 6 and Proposition 11.

### **Theorem 7**

*Let* $\beta \in S_n$ *be a transposition. Then*

1. $$c(0, \beta )=2(n-2)!$$, $$n >1$$.
2. $$c(3,\beta )=4(n-2)(n-2)!$$, $$n >1$$.
3. $$c(4,\beta )=(n-2)(n-3)(n-2)!$$, $$n >2$$.
4. $$c(k, \beta )=0$$, for $$5\le k\le n$$.

Formulas (1), (2) and (3) in previous proposition coincide with the number of permutations of *n* symbols, with $n > 1$, having exactly 2, 3 and 4 points, respectively, on the boundary of their bounding square (that are labeled as sequences A208529, A208528 and A098916 in the OEIS database, respectively). Details about this definitions can be consulted in Deutsch (2012). Therefore, our result provides another interpretation for these sequences in the OEIS database.

Now we give a formula for $c(k, \beta )$ when $\beta$ is any fixed-point free involution. Let *a*(*n*) be the “number of deranged matchings of 2*n* people with partners (of either sex) other than their spouse” (sequence A053871).

### **Theorem 8**

*Let* $\beta \in S_{2m}$ *be a fixed-point free involution*, $m \ge 2$. *Then*

1. $c(k, \beta )=0$, for *k* and odd integer,
2. $$c(k, \beta )=2^{m}m!\left( {\begin{array}{c}m\\ j\end{array}}\right) a(j)$$, for $$k=2j$$, $$j=0, 1, 2, \dots $$

### *Proof*

By hypothesis, the cycle decomposition of $\beta$ consists of exactly *m* transpositions. From Proposition 3 we have that if $\alpha$ does not commute on one point in a transposition $\beta _1$ of $\beta$ then $\alpha$ does not commute on the two points in $\beta _1$, therefore any permutation does not commute with $\beta$ in a even number of points, which implies that $c(k, \beta )=0$ for *k* odd. Now, if $k=2j$, we obtain all the permutations $\alpha$ that *k*-commutes with $\beta$ and that do not commute with $\beta$ on exactly *j* transpositions, of $\beta$. There are $$\left( {\begin{array}{c}m\\ j\end{array}}\right) $$ ways to select a set, say $\{\beta _1, \dots ,\beta _j\}$, of *j* transpositions of $\beta$. Let $X=\bigcup _{i=1}^j\mathrm{set}(\beta _i)$. By Proposition 4, there exists exactly *j* transpositions, $\beta _1', \dots , \beta _j'$ of $\beta$ such that $\alpha (X)=X'$, where $X'=\bigcup _{i=1}^j\mathrm{set}(\beta _i')$. Given a selection of cycles $\{\beta _1, \dots ,\beta _j\}$, there are $$\left( {\begin{array}{c}m\\ j\end{array}}\right) $$ ways to select a set, $\{\beta _1', \dots , \beta _j'\}$, of *j* transpositions of $\beta$ that will satisfy $\alpha (X)=X'$. We construct $\alpha |_X$ as follows: First, we define the auxiliary bijection from *X* onto $X'$ as

3 $$\begin{aligned} \alpha '|_X=\left( \begin{array}{c} B_1 \\ B'_{i_1} \\ \end{array} \right) \dots \left( \begin{array}{c} B_j \\ B'_{i_j}\\ \end{array} \right) ; \end{aligned}$$

where $B_l$ (resp. $B_l'$) is an improper block of the cycle $\beta _l$ (resp. $\beta _l'$), for every $l \in \{1, \dots , j\}$, and $\{B'_{i_1}, \dots , B'_{i_j}\}=\{B_1', \dots , B_j'\}$. There are $2^j$ ways to select the first element in each of the blocks $B_1, \dots , B_j$. There are *j*! ways to arrange the blocks $B_1'$, ..., $B_j'$, in the second row of (3). Until this step $\alpha '|_X$ is a bijection from *X* onto $X'$ that commutes with $\beta$ on the cycles $\beta _1, \dots , \beta _j$. We can think that every block $B_i'$ is a partner $B_i'=xy$. We construct $\alpha |_X$ from $\alpha '|_X$ by re-pairing the elements in the blocks $B_{i_1}', \dots , B_{i_j}'$ in such a way that every point is paired with a point other than its original partner, this can be made in *a*(*j*) ways. Finally, there are $(m-j)!2^{m-j}$ ways to construct $\alpha |_{[2m]\setminus X}$ in such away that it commutes with $\beta$ on the $m-j$ transpositions of $\beta$ not in $\{\beta _1, \dots ,\beta _j\}$. Therefore

$$\begin{aligned} 2^j j!a(j)\left( {\begin{array}{c}m\\ j\end{array}}\right) ^2 \frac{(m-j)!}{2^j}2^m=2^mm! \left( {\begin{array}{c}m\\ j\end{array}}\right) a(j). \end{aligned}$$

$$\square $$

### **Theorem 9**

*Let* $\beta \in S_{2m}$ *be a fixed-point free involution. Then*

$$\begin{aligned} \sum _{m, j \ge 0} c(2j, \beta ) \frac{z^m}{m!}\frac{u^j}{j!}= \left( \left( 1-2\,z \right) \sqrt{1-4\,{\frac{zu}{1-2\,z}}} \exp \left( \frac{2zu}{1-2\,z}\right) \right) ^{-1}. \end{aligned}$$

### **Sketch**

We use the well-known EGF for *a*(*n*) [see the formula section of sequence A053871 in Sloane (2015)]

$$\begin{aligned} \sum _{n \ge 0} a(n) \frac{x^n}{n!}=\left( \exp (x)\sqrt{1-2x}\right) ^{-1}, \end{aligned}$$

and the result follows by using standard techniques of bivariate generating functions similarly as in the proof of Theorem 4. $\square$

## Conclusions

In this paper we give some techniques to work with *k*-commuting permutations. We present some formulas for the number of permutations that *k*-commute with $\beta$, when $\beta$ is any permutation and $k\le 4$. Also we obtain formulas for $c(k, \beta )$ when $\beta$ is either a transposition, or an *n*-cycle, or a fixed-point free involution, for any *k*. These results could be useful when we work in problems related with almost commuting permutations. Even more, these enumerative results could be useful to find relations between integer sequences in the OEIS database, as Rivera (2015) showed.

The problem of computing in an exact way the number $c(k, \beta )$ could be a difficult task. However, it is possible that for some specific cycle type of permutations, the problem can be managed. We leave as an open problem to find another technique, or a refinement of the presented in this article, to compute $c(k, \beta )$ in exact way, or at least to obtain non trivial upper and lower bounds for this number.
